# Genome Sequence of Flavor-Producing Yeast Saprochaete suaveolens NRRL Y-17571

**DOI:** 10.1128/MRA.00094-19

**Published:** 2019-02-28

**Authors:** Hana Lichancová, Viktória Hodorová, Karolina Sienkiewicz, Sarah Mae U. Penir, Philipp Afanasyev, Dominic Boceck, Sarah Bonnin, Siras Hakobyan, Pawel S. Krawczyk, Urszula Smyczynska, Erik Zhivkoplias, Maryna Zlatohurska, Adrian Odrzywolski, Eugeniusz Tralle, Alina Frolova, Leszek P. Pryszcz, Broňa Brejová, Tomáš Vinař, Jozef Nosek

**Affiliations:** a Department of Biochemistry, Faculty of Natural Sciences, Comenius University in Bratislava, Bratislava, Slovak Republic; b Faculty of Mathematics, Informatics and Mechanics, University of Warsaw, Warsaw, Poland; c Philippine Genome Center, National Science Complex, University of the Philippines Diliman, Quezon City, Philippines; d Laboratory of Evolutionary Genomics, Vavilov Institute of General Genetics, Moscow, Russia; e Algorithms in Bioinformatics, ZBIT Center for Bioinformatics, University of Tübingen, Tübingen, Germany; f Center for Genomic Regulation (CRG), Barcelona Institute of Science and Technology, Barcelona, Spain; g Institute of Molecular Biology NAS RA, Yerevan, Armenia; h Laboratory of RNA Biology and Functional Genomics, Institute of Biochemistry and Biophysics, Polish Academy of Sciences, Warsaw, Poland; i Department of Biostatistics and Translational Medicine, Medical University of Lodz, Lodz, Poland; j Biology Education Centre, Uppsala University, Uppsala, Sweden; k Institute of Microbiology and Virology, National Academy of Science of Ukraine, Kyiv, Ukraine; l Medical University in Lublin, Lublin, Poland; m International Institute of Molecular and Cell Biology in Warsaw, Warsaw, Poland; n Institute of Molecular Biology and Genetics, National Academy of Sciences of Ukraine, Kyiv, Ukraine; o Department of Computer Science, Faculty of Mathematics, Physics and Informatics, Comenius University in Bratislava, Bratislava, Slovak Republic; p Department of Applied Informatics, Faculty of Mathematics, Physics and Informatics, Comenius University in Bratislava, Bratislava, Slovak Republic; Broad Institute of MIT and Harvard University

## Abstract

Saprochaete suaveolens is an ascomycetous yeast that produces a range of fruity flavors and fragrances. Here, we report the high-contiguity genome sequence of the ex-holotype strain, NRRL Y-17571 (CBS 152.25). The nuclear genome sequence contains 24.4 Mbp and codes for 8,119 predicted proteins.

## Abstract (precis)

Saprochaete suaveolens is an ascomycetous yeast that produces a range of fruity flavors and fragrances. Here, we report the high-contiguity genome sequence of the ex-holotype strain, NRRL Y-17571 (CBS 152.25).

## ANNOUNCEMENT

Saprochaete suaveolens is a fermentative yeast from the *Magnusiomyces*/*Saprochaete* clade (phylum *Ascomycota*, subphylum *Saccharomycotina*). It has been isolated from nutrient-rich sources, including industrial wastes, brewery water, process water from wheat-starch production plants, effluent milk, maize mash, soybean flakes, figs, and dragon fruits, and some strains were isolated from patients with pulmonary infections (1–3). It produces large amounts of volatile organic compounds with an intensive fruity odor (3–5).

The S. suaveolens strain NRRL Y-17571 was originally isolated from water in a brewery (1). Its genome was assembled by the combination of long reads (MinION, Oxford Nanopore Technologies) and short reads (HiSeq 2000, Illumina). DNA was isolated from a culture grown overnight in yeast extract-peptone-dextrose (YPD) medium (1% [wt/vol] yeast extract, 2% [wt/vol] peptone, 1% [wt/vol] glucose) at 28°C using a standard protocol and purified using the DNeasy mini spin column (Qiagen) for HiSeq 2000 analysis or Genomic-tip 100/G (Qiagen) for MinION analysis (6). Total cellular RNA from the midexponential phase culture grown in yeast extract-peptone-galactose (YPGal) medium (1% [wt/vol] yeast extract, 2% [wt/vol] peptone, 2% [wt/vol] galactose) at 28°C was extracted with hot acidic phenol (7) and purified with the RNeasy minikit (Qiagen).

We obtained 204,824 long reads (mean, 9,011 nucleotides [nt]; longest read, 211,620 nt) totaling 1.8 Gbp (∼74× coverage) with a MinION Mk-1B device on a R9.4.1 flow cell with a SQK-LSK109 kit and base called with ONT Albacore (v. 2.3.1). A paired-end (2 × 101 nt) TruSeq PCR-free DNA library was sequenced on a HiSeq 2000 platform in Macrogen, Korea, which yielded 64,378,402 reads (6.4 Gbp, ∼262× coverage). RNA-Seq was performed with NovaSeq 6000 system in Macrogen, Korea, which yielded 42,932,052 reads from a TruSeq mRNA V2 nonstranded paired-end (2 × 101 nt) library.

Table 1 presents candidate genome assemblies. The final assembly is based on miniasm, which had the smallest number of contigs and did not show apparent assembly artifacts. To further improve this assembly, we removed contigs containing fragments of mitochondrial DNA (mtDNA) and rRNA genes, individually polished rRNA gene repeats, and replaced regions upstream and downstream of rRNA gene repeats with 505 bp from DBG2OLC and 309 bp from Canu assemblies, respectively. The nuclear genome has a GC content of 39.5% and likely consists of at least 7 chromosomes, because both ends of 4 contigs and one end of 6 contigs are terminated by telomeric repeats with a predominant motif CA_3_G_5-7._ About 2% of the genome (508 kbp) is covered by simple and low-complexity repeats identified with RepeatMasker v. 4.0.7 (8).

**TABLE 1:** Candidate genome assemblies^*a*^

Assembly software	Software version	Polishing procedure	Length of assembly (Mbp)	No. of contigs	No. of contigs >50 kbp	Longest contig	*N*_50_ value	No. of mismatches per 100 kbp	No. of indels per 100 kbp
SPAdes (18)	3.12.0		24.2	2,224	137	640 kbp	173 kbp		
Canu (19)	1.7.1	Pilon (2×)	24.9	26	24	4.2 Mbp	1.7 Mbp	41.7	13.9
MaSuRCA (20)	3.2.8	Pilon (1×)	25.4	29	23	6.8 Mbp	2.7 Mbp	45.7	7.6
Miniasm (21)/minimap2 (22)	0.3/2.12	Racon (2×)	24.5	15	13	3.7 Mbp	2.8 Mbp	81.5	367.9
DBG2OLC (23)/platanus (24)	1.2.4	Racon (1×), Pilon (1×)	25.8	31	24	4.2 Mbp	1.8 Mbp	55.2	35.1
Final		Racon (2×), Pilon (2×)	24.4	12	11	3.8 Mbp	2.8 Mbp	45.6	11.0

a Statistics were produced with Quast v. 4.5 (15). To estimate mismatches and indels, SPAdes assembly based on Illumina short reads was used as a reference. With SPAdes, the result was filtered for length >100 and coverage >10. Canu assembly used only reads overlapping SPAdes by >200 bp, and we filtered out contigs supported by fewer than 5 reads. All assemblies were polished with Pilon v. 1.21 (16) and Racon v. 1.3.1 (17). Most of the size differences between candidate assemblies can be accounted for by mtDNA and rRNA gene fragments as well as other repetitive sequences.

RNA-Seq reads processed with Trimmomatic v. 0.36 (9) were assembled into transcripts with Trinity v. 2.8.3 (10). We trained Augustus v. 3.2.3 (11) on the Magnusiomyces capitatus data set (12) and, using RNA-Seq transcripts aligned to the reference with blat v. 34 × 1 (13), we predicted 8,119 protein-coding genes.

The genome sequence of S. suaveolens will provide a basis for understanding metabolic pathways involved in the production of volatile organic compounds, suitable as flavors and aromas in the food industry, and genetic traits associated with the ability to colonize humans.

### Data availability.

This whole-genome shotgun assembly has been deposited in EMBL ENA under the accession no. CAAAMA010000000. Illumina, MinION, and RNA-Seq reads have been deposited under accession no. ERR3039972, ERR3040055, and ERR3039974, respectively. Genome annotations are available through a genome browser at http://genome.compbio.fmph.uniba.sk/ and are also archived through Zenodo (14).
